# Radiographic markers of hip dysplasia in young adults: predictive effect of factors in early life

**DOI:** 10.1186/s12891-023-06199-y

**Published:** 2023-02-11

**Authors:** Lene B. Laborie, Stein Atle Lie, Karen Rosendahl

**Affiliations:** 1 Department of Clinical Medicine, University of Bergen, Bergen, Norway; 2 Department of Radiology, Section for Pediatric Radiology, Haukeland University Hospital, Jonas Lies vei 65, 5021 Bergen, Norway; 3 Department of Clinical Dentistry, University of Bergen, Bergen, Norway; 4 The Norwegian Arthroplasty Register, Haukeland University Hospital, Bergen, Norway; 5 Department of Clinical Medicine, UiT the Arctic University of Norway, Faculty of Health Sciences, Tromsø, Norway; 6 Section of Paediatric Radiology, University Hospital of North Norway, Tromsø, Norway

**Keywords:** Ultrasound, Sonography, Hip dysplasia, Risk factors, Acetabular dysplasia

## Abstract

**Background and objectives:**

Acetabular dysplasia in young adults occurs, despite screening for developmental hip dysplasia (DDH) in the neonatal period. We aimed to examine how early life factors predict radiographic measurements of acetabular dysplasia at 18–19 years of age.

**Methods:**

From a previous randomized trial (*n* = 12,014; 1988–90) evaluating the role of hip ultrasound in newborn screening of DDH, 4469 participants (2193 males) were invited to a follow-up 18 years later (2007–09), of which 2370 (53% attendance; 932 males) met. We examined associations between early life factors and four radiographic measurements for acetabular dysplasia at skeletal maturity. Hierarchical regressions, with addition of variables observed/measured consecutively in time, were analyzed using mixed effects models considering hip as the unit in the analyses. The study is approved by the Regional Ethics Committee.

**Results:**

In total, 2340 participants (921 boys), mean age 18.7 years, (SD 0.6) had hip radiographs performed at follow-up and were included. Early life factors significantly predicting radiographic acetabular dysplasia at age 18–19-years included female gender, breech, low acetabular inclination (alpha) angle and sonographic instability, abduction treatment, as well as the velocity of growth during childhood. A positive family history of DDH was not associated with acetabular dysplasia at skeletal maturity.

**Conclusion:**

The acetabular inclination (alpha) angle as measured on ultrasound at birth turned out to be a significant predictor of dysplasia at 18–19 years of age. The discordant role of a positive family history in early versus adult hip dysplasia is intriguing, warranting further studies on the genetic mechanisms of DDH.

**Supplementary Information:**

The online version contains supplementary material available at 10.1186/s12891-023-06199-y.

## Introduction

Developmental dysplasia of the hip (DDH) is a common pediatric musculoskeletal disorder, with a neonatal prevalence reported up to 4%, more often in newborn girls than in boys, and the left hip more often affected than the right [1–4]. The condition is characterized by a shallow or dysplastic hip socket, with risks of developing progressive joint dislocation in severe cases. When detected in the newborn period, DDH is typically treated non-invasively with an abduction device for 3–4 months. Management of late detected cases of DDH is more challenging, and severe cases may require major, invasive surgery. Acetabular dysplasia can occur in early adulthood as residual dysplasia despite early detection and treatment of DDH, or because of late- or undetected disease. The prevalences of acetabular dysplasia at skeletal maturity reported in the literature vary greatly. In this follow-up cohort, the prevalence has previously been reported as ranging from three to 19%, depending on the radiographic measurement used, and with similar variations in other studies [5–7]. Acetabular dysplasia in young adults predisposes to osteoarthritis and serious functional disability, often necessitating total hip replacement [8]. A large study from 2005 indicated that subjects with hip osteoarthritis following hip dysplasia might be younger at time of onset of osteoarthritis than subjects with hip osteoarthritis in normal hips [3]. Perinatal risk factors known to be associated with DDH in newborns are female gender, breech presentation at delivery, and a positive family history of DDH [9–11]. Associations with radiographic acetabular dysplasia at 18–19 years are incompletely understood. We here aim to examine if early life factors, including perinatal risk factors, clinical and ultrasound findings, and DDH treatment, predict radiographic markers of acetabular dysplasia at 18–19 years of age.

## Materials and methods

**Fig. 1:**
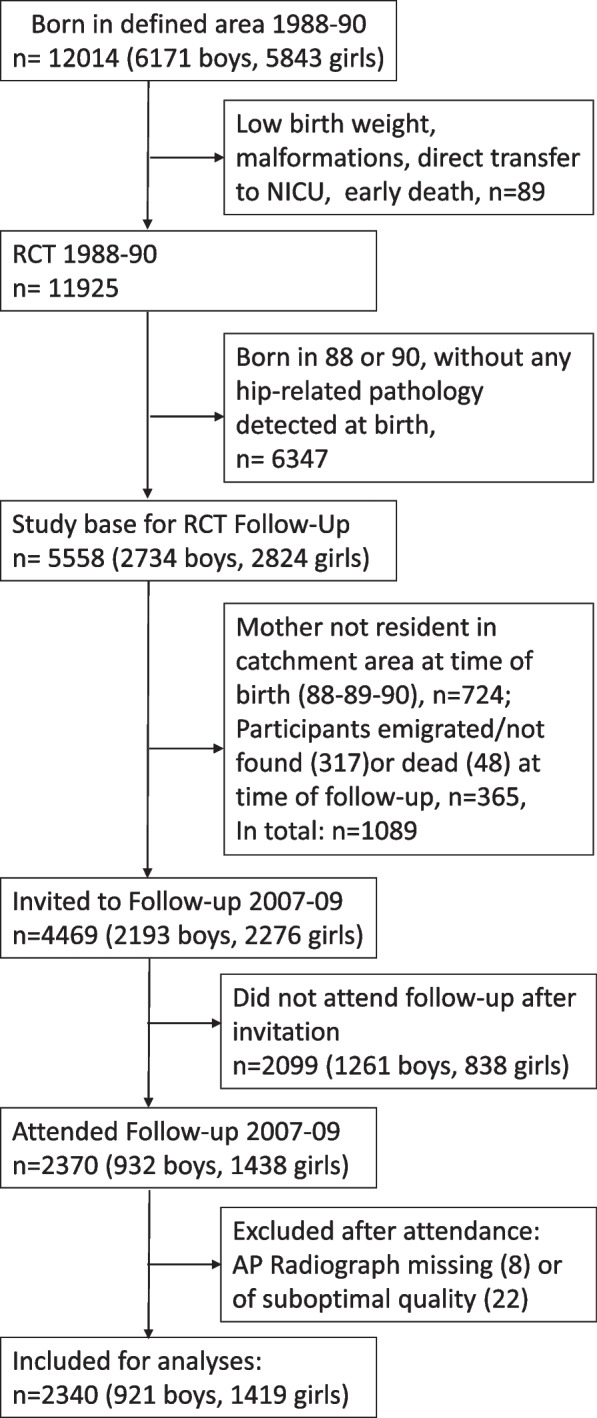
The 11,925 newborns included in the original RCT (1988–90) were randomly assigned to one of three neonatal screening groups: universal ultrasound (*n* = 3613); selective ultrasound (*n* = 4388); no ultrasound (*n* = 3924). The 2340 individuals included for analyses at follow-up (2007–09) originated equally from the three original screening groups (*n* = 776, *n* = 745, *n* = 819, respectively)

In total 12,014 (6171 boys, 5843 girls) babies were born at the university hospital from January 1988 through June 1990, and eligible for enrollment in a large initial RCT evaluating the role of neonatal hip ultrasound in newborn screening of DDH (Fig. 1). Of the 11,925 individuals included in the initial RCT, in total 4469 (2193 boys, 2276 girls) were invited to a smaller follow-up around 18 years later, including all participants born in 1989, as well as all participants with a pathological neonatal hip ultrasound born in 1988 or 1990. Of the 4469 invited, 2370 (53% attendance, (932 boys, 1438 girls) met. The RCT and the follow-up study are described in details in previous papers [12, 13].

### Data collected in the neonatal period

#### Perinatal risk factors

Information on gender, breech position at delivery, and status of newborn clinical hip examination was recorded and available for all follow-up participants, and did not differ significantly across the three screening groups [13]. In total 121 (32 males, 89 females) of the 2340 participants (5.2%) were recorded as breech position at time of delivery. Details on family history of DDH were recorded for all the 1521 follow-up participants originating from the RCT ‘universal ultrasound’ (*n* = 776) and ‘selective ultrasound’ (*n* = 745) groups, but not for those in the ‘no ultrasound’ (*n* = 819) group. In total 186 (71 males, 115 females) of these 1521 (12.2%) had a positive family history of DDH, as at least one first grade relative or at least two second-grade relatives with DDH. A positive family history was associated with both treatment in the neonatal period and with late treatment onset after one month of age (Chi square tests, *p* < 0.001 in both cases).

#### Clinical hip joint instability

Every newborn received a hip examination within 2–3 days of life, by a designated group of pediatricians with at least two years of experience. Each hip was judged to be stable, pathologically instable, dislocatable (positive Barlow test) or dislocated (positive Ortolani test). Cases with a dislocatable or dislocated hip, confirmed by a senior pediatrician, led to immediate abduction treatment. A persistent pathologically instable (but not dislocatable) hip, confirmed by the senior pediatrician within the following two days, was re-evaluated after six weeks.

#### Pathological morphology and/or instability on newborn hip ultrasound

All newborns from the universally screened group, and all high-risk newborns from the selectively screened group, received a single examiner hip ultrasound at birth (948 in total; 349 boys, 599 girls). This modified Graf’s method (Rosendahl) evaluate acetabular morphology and hip joint stability separately, based on the acetabular inclination angle (α-angle) and a Barlow manoeuver, respectively [14, 15].

##### Algorithm

for ultrasound follow-up and for treatment

Indications for immediate neonatal abduction treatment were persistent dislocatable/dislocated hips on a repeated, single-examiner clinical examination, or severe sonographic dysplasia (α < 43°) irrespective of clinical and sonographic instability. Hips with a mildly dysplastic morphology (43° ≤ α < 50°) were treated if they were also clinically or sonographically dislocatable/dislocated. Sonographically immature (50° ≤ α < 60°) or mildly (43° ≤ α < 50°) dysplastic but clinically stable hips had sonographic and clinical surveillance every fourth week until normalization or until treatment was instigated due to lack of improvement. Routines for abduction treatment included a Frejka’s pillow splint from birth until around three to four months of age, followed by an age-adapted orthosis if required.

In total 220 (32 boys, 188 girls) received abduction treatment, of which 202 (31 boys, 171 girls) received early treatment, whereas 18 (1 boy, 17 girls) were detected late, i.e. after one month of age [16, 17]. The 18 late cases ranged from sonographically verified acetabular dysplasia without any displacement of the femoral head (13 cases), to severe cases of subluxated [3] or fully dislocated [2] femoral heads. In all the late detected cases, an age-adapted, late abduction treatment (Frejka’s pillow or orthosis) was instigated. All the five severe cases, i.e. those with subluxation or dislocation, received additional traction treatment, followed by cast and/or orthotic treatment. The two dislocated cases also had a closed reduction performed.

### Data collected at child health clinics during childhood

Weight was measured at six weeks of age, at four, five, six, 10, 11 and 18 months, and at two, four, six, eight and 12 years. Length was measured at six and 18 months, and height at two, five, six and 12 years. These anthropometric data were available for 1849 (761 males, 1088 females) of the maturity review participants. Weight and height were also recorded at skeletal maturity.

### Data collected at maturity review, age 18–19 years: Radiographic protocol, evaluation of images, and radiographic measurements for acetabular dysplasia

**Fig. 2:**
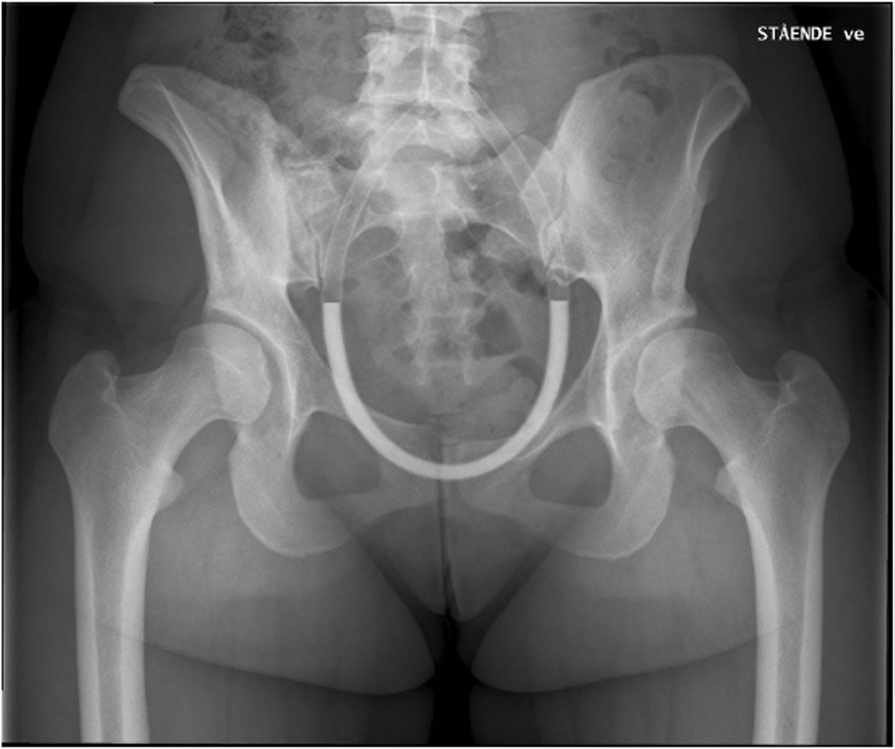
A weight-bearing anteroposterior radiograph of a study participant at skeletal maturity, revealing bilateral moderate dysplasia. The film/focus distance was 1.2 m and centered at 2 cm proximal to the symphysis, and hips were kept in a neutral abduction–adduction position with toes pointing forwards

**Fig. 3:**
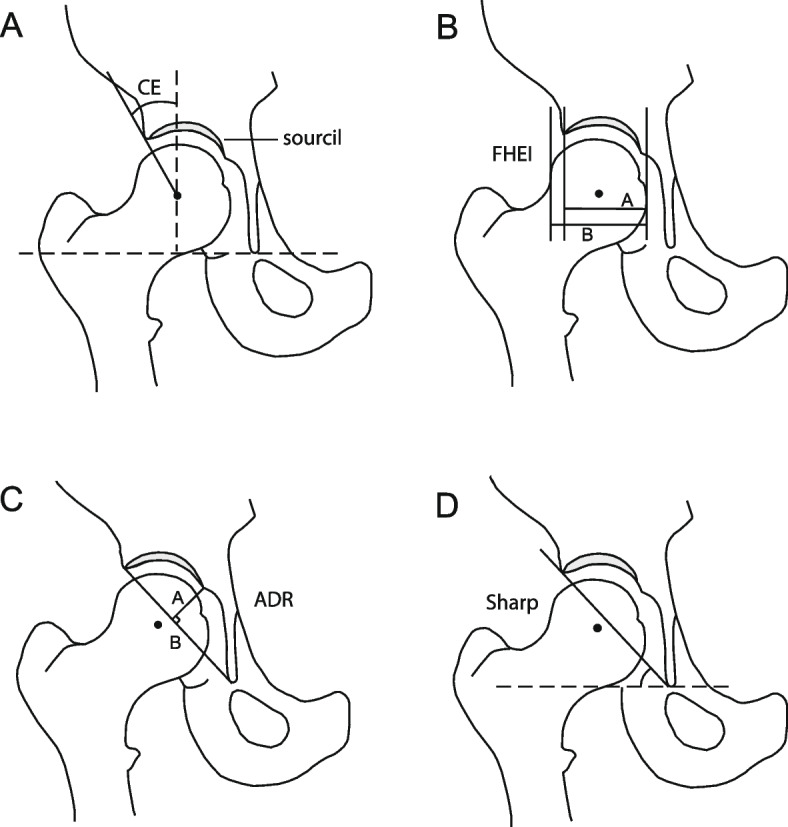
Measurements describing the position of the femoral head relative to the acetabular cavity: Centre edge (CE) angle of Wiberg (**A**), and Femoral head extrusion index (FHEI) (**B**). Measurements describing the acetabular anatomy: Acetabular depth-width ratio (ADR, (A/B)*1000) (**C**), and Sharp’s angle (**D**)

Exclusion criteria at follow-up included missing radiographs due to uncertain pregnancy status or to refusal of the radiographic exam [8]. and radiographs of suboptimal technical quality [22]. defined as excessive pelvic rotation assessed by a foramen obturator index outside the range 0.6 to 1.8 [18]. The weight-bearing, anteroposterior (AP) view (Fig. 2) was obtained according to a standardized protocol, by one specifically trained radiographer, with a low-dose digital radiography technique [19]. The radiographs were measured in a digital measurement program by three trained examiners [20]. Detailed descriptions of the digital measurement program, of its accuracy and of the measurements included have been reported previously [21]. As there is no clear consensus on the definition of acetabular dysplasia at skeletal maturity, the four most common radiographic measurements associated with acetabular dysplasia were assessed (Fig. 3a-d) [5, 19].

### Ethics

The research protocol, including analyses of the non-responders, was approved by the Regional Ethical Committee for Medical and Health Research (REK 20594). The protocol was registered at ClinicalTrialsGov (NCT01818934, 21th of March 2013). All participants of the follow-up study gave written informed consent according to the 1964 Declaration of Helsinki.

### Statistical analysis

**Fig. 4:**
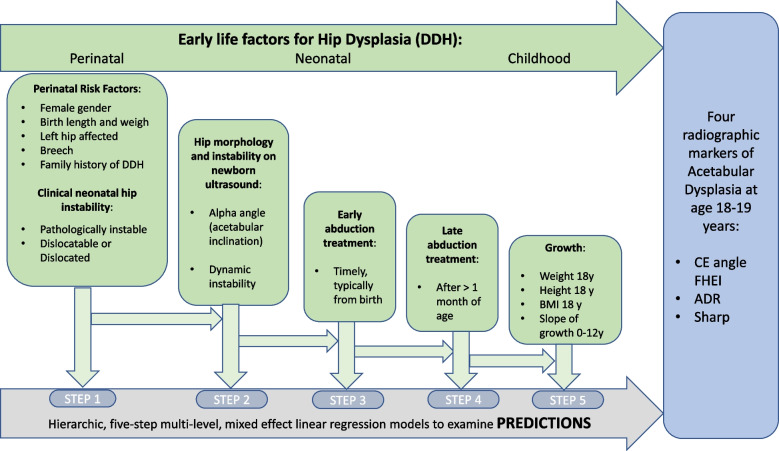
The statistical five-step hierarchic model used to analyze how early life factors can predict four radiographic measurements of acetabular dysplasia at 18–19 years of age. DDH Hip dysplasia. CE angle Center edge angle of Wiberg. FHEI Femoral head extrusion index. ADR Acetabular depth-width ratio. Y years old

For simple comparisons of categorical variables, chi-square tests were applied, while t-tests were applied for continuous variables. To study the influence of the early life factors, we applied hierarchic (in five steps) regression models. In these, we added factors in a chronological order, corresponding to the age at which these factors were observed/measured (Fig. 4).

To take into account that each hip was measured separately, we applied mixed effects models using hip as the unit in the analysis. Separate models were set up for each of the four radiographic measurements for acetabular dysplasia at skeletal maturity. Since the anthropometric measures height/length, weight and body mass index (BMI) were measured repeatedly during childhood until 12 years of age, we calculated slope of growth using linear regressions, for each individual separately, and used these slopes as predictors in the final regressions. To compare the quality of the five different steps in the hierarchical regressions, Akaike information criterion (AIC) was reported. Furthermore, intra class correlations (ICCs) for the correlation between right and left hip for each of four radiographic measurements were reported. As a sensitivity analysis, inverse probability weights (IPW) were applied in the final model to account for the sampling from those from the original RCT who were invited to the maturity review. The IPW was calculated based on the predicted probabilities from logistic regression models including variables (sex, birth weight, breech position, year of birth, randomization group, and abduction treatment) known to be related to sampling from the original RCT to the present data. Since some variables had missing data, we included missing indicators to exploit the full dataset in the predictive models [22]. The analyses were performed using Stata version 17 (College Station, TX, USA). *P*-values less than 0.05 (5%) were considered statistically significant.

## Results

Of the 4469 subjects invited to the maturity review, 2370 (53%) attended, of which 2340 (1419 (61%) female participants), were included for further analysis (corresponding to 4680 hips). Mean age of participants at follow-up was 18.7 years (SD 0.6). Mean weight, height and BMI of participants at follow up were 63.6 kg (SD 11.9), 166.3 cm (SD 6.1) and 23.0 kg/m^2^ (SD 3.9) for females and 76.0 kg (SD 14.0), 180.2 cm (SD 6.8) and 23.4 kg/m^2^ (SD 4.0) for males, respectively. The distribution of the radiographic measurements for acetabular dysplasia according to gender and side at skeletal maturity has been reported for a sub-group of the cohort in previous papers [5, 19].  The prevalence of pathological values for the four radiographic markers suggestive of hip dysplasia among the 2340 participants were as follows, reported for the right hip, for females and males, respectively: 3.1% and 1.9% had a Wiberg CE-angle < 20˚; 5.1% and 3.8% had a Femoral Head Extrusion index (FHEI) < 75%; 9.1% and 8.5% had a Acetabular Depth Ratio (ACR) < 250; and 11.7% and 4.6% had a Sharp angle > 45˚. Furthermore, 15.0% of females had one of the four findings on the right side, and 2.7%, 1.8% and 0.9% of females had two, three, or all four findings, respectively. Corresponding figures for males were 11.5%, 1.6%, 0.9% and 0.3%, respectively. Similar findings were found for the left side.

For the center-edge (CE) angle of Wiberg, the most widely used radiographic measurement for acetabular dysplasia, we found for the demographic variables entered in step 1, that gender, breech position, and side, but not a positive family history, were significant predictors (all* p* < 0.05) (Table 1a). In step 2 we found that the alpha angle measured at birth also contributed significantly to the prediction (*p* < 0.001). Late abduction treatment, added in step 4, also contributed to the prediction (*p* = 0.003), while changes in the anthropometric measures had no additional effect. We also observed that there were only minor changes in the predictors added in a previous step, except for hip instability on ultrasound, which was nearly significant in step 2 (*p* = 0.060), but became significant when abduction treatment was added in step 3 (all p < 0.05). Clinical instability at birth had no predictive effect on the CE angle (*p* = 0.207).

For the femoral head extrusion index (FHEI), we found that side and pathological clinical instability were significant predictors in step 1 (both *p* < 0.05) (Table 1b). In step 2, the alpha angle and hip instability on ultrasound were both significant predictors, while this also was the case for early abduction treatment in step 3 and late abduction treatment in step 4 (all *p* < 0.05). Change in weight but not height was significant in step 5 (*p* < 0.05).

For the ADR, the demographic variables gender, breech position and side entered in step 1 were significant predictors (all *p* < 0.05), while clinical instability was not (Table 1c). Neither the alpha-angle and hip instability on ultrasound entered in step 2, nor early abduction treatment entered in step 3 were significant predictors, while late abduction treatment entered in step 4 was (*p* = 0.018). The variables for growth, i.e. change in anthropometrics added in step 5 had no significant prediction on ADR (all *p* > 0.05).

For the Sharp’s angle, gender entered in step 1, and the alpha angle entered in step 2 were significant predictors (both *p* < 0.05) (Table 1d). Furthermore, change in height and weight were significant in step 5 (both *p* < 0.05). We observed that adding new variables to the models had little impact on the variables entered in an earlier step.

Only minor changes to the final results were detected in the fully adjusted IPW-model (Table 2). These changes included a non-significant coefficient for Hip instability on ultrasound (*p* = 0.075) for the center-edge (CE) angle of Wiberg, a non-significant coefficient of slope of weight (*p* = 0.064) for FHEI, and a non-significant coefficient for the Alpha angle (0.072) for Sharp’s angle. For ADR the IPW-analysis were equal to the analysis without IPW. The intra class correlation between right and left hip was 0.39 (for Sharp’s angle), 0.63 (for FHEI), 0.69 (for Wiberg) and 0.71 (for ADR).

## Discussion

Early life factors significantly predicting one or several radiographic markers for acetabular dysplasia at age 18–19-years included female gender, left hip involvement, breech presentation, lower newborn acetabular inclination (alpha) angle and instability on ultrasound, early abduction treatment, late onset of abduction treatment following detection of DDH after one month of age, as well as the velocity of height and weight growth during childhood. Of note is that a positive family history of DDH did not predict acetabular dysplasia at skeletal maturity.

In contrast to several previously published studies reporting on risk factors for neonatal DDH, there are few reports addressing these risk factors at skeletal maturity. Sink et al. conducted a study to identify the prevalence of risk factors for DDH that would have warranted selective ultrasound neonatal screening in patients with symptomatic acetabular dysplasia after skeletal maturity [23]. The authors evaluated several risk factors for DDH including gender, family history of hip osteoarthritis or DDH, breech, method of delivery, birth order, and previous hip treatments, in 68 (67 females) skeletally mature patients (average age 26.4 [range 13–47] years) undergoing corrective osteotomy for symptomatic acetabular dysplasia. They found that only 2.9% of the patients had a positive family history of DDH, whereas 36.8% of the patients had a family history of hip osteoarthritis in a first- or second degree relative (but not confirmed by the family as DDH). They also reported that 98.5% and 11.8% of the patients were females and breech, respectively.

The exact role of family history is not fully understood. A positive family history of DDH is a common reason for referral to neonatal hip ultrasound, and has been shown to be of importance to neonatal DDH, also in our cohort [10]. However, an evidence synthesis for the American Academy of Pediatrics’ (AAP) clinical practice guideline for the early detection of DDH, odds ratios (OR) for DDH given different risk factors were investigated. The OR of 1.7 for DDH among those with a positive family history was not statistically significant (95% CI 0.05–55) [11]. These findings highlight the need to better understand the genetic influence on both neonatal DDH, and acetabular dysplasia at skeletal maturity.

In our study, a positive family history as recorded in the neonatal period, did not significantly predict any of the radiographic markers for mature acetabular dysplasia. This is interesting, especially since the family history is associated with DDH in the neonatal period in this same cohort. One explanation might be that a positive family history elicited a hip ultrasound, followed by abduction treatment in those testing positive. Thus, one might speculate that when detected at birth, DDH in these children can be successfully treated, without a sequela seen radiographically at skeletal maturity. One could also speculate that acetabular dysplasia at skeletal maturity might be genetically different from the developmental dysplasia diagnosed in the neonatal period.

Breech turned out to be a significant predictor for the CE angle and the acetabular depth ratio (ADR) at skeletal maturity, supporting previous findings that mechanical, intrauterine factors play an important role for development of the acetabulum [9–11].

As for the neonatal ultrasound examination, we found that the acetabular inclination (alpha) angle in particular, but also sonographic assessment of hip instability, significantly predicted radiographic markers for hip dysplasia at skeletal maturity. Of note is that clinically assessed hip instability did not, thus supporting previous reports on the method’s low sensitivity and specificity [24].

Interestingly, our study showed that the velocity of height and weight growth during childhood was a significant predictor for the acetabular inclination, as measured by Sharp’s angle at skeletal maturity. The acetabulum develops from three bones; the ilium, ischium and pubis, separated by the cartilage complex [25]. This complex is triradiate shaped medially and cup shaped laterally, allowing the acetabular cavity to expand during growth until it closes at around 12 years of age in females and 14 years in males [26]. Appositional growth at the periphery of the hip socket further increases its depth, whilst the acetabular concavity develops in response to the presence of the spherical femoral head. The weight and height measurements in the present study were registered until 12 years of age, thus before complete closure of the triradiate cartilage. Our results suggest that increased compressive stresses to the acetabular roof during weight bearing activities might have influenced acetabular inclination at skeletal maturity. Further details related to this finding will be presented elsewhere.

We acknowledge several limitations to our study. The attendance rate for the maturity review was 53%. We have previously reported analyses from this follow-up study that show no differences between the responders and the non-responders, except for the gender distribution [12, 27]. We speculate that the skewed gender distribution in the recall cohort, i.e. more females than males attending, might be influenced by the fact that there was a higher rate of females treated for DDH in the newborn period as compared to males. Another limitation includes the lack of a strict definition of acetabular dysplasia at skeletal maturity. We have therefore chosen to include the four most widely used radiographic measurements. We have previously reported varying prevalence of acetabular dysplasia at age 18–19 years in a population-based sub-group of our study cohort, ranging from 3 to 19% depending on which measurement used, as well as only moderate overlap between the four measurements [5]. To adjust for the attendance rate, we applied inverse probability weights (IPW). We found that these analyses correspond to the analyses without IPW. Strengths of our study include a strict RCT protocol, few pediatricians performing the newborn clinical hip examinations, and a single, highly experienced examiner performing the newborn hip ultrasound throughout the whole study period. The radiographic measurements for acetabular dysplasia at skeletal maturity were performed in a validated, digital program, by only three examiners.

## Conclusion

Early life factors that significantly predicted one or several radiographic measurements for acetabular dysplasia at age 18–19 years included female gender, breech position, lower newborn alpha angles and hip-instability on ultrasound, early abduction treatment, late onset of abduction treatment following detection of DDH after one month of age as well as the velocity of growth during childhood. Clinical hip instability was only associated with the femoral head extrusion index. A positive family history of DDH was, however, not significantly associated with any of the radiographic measurements for acetabular dysplasia at skeletal maturity. Further understanding of the genetic contribution to DDH in childhood and as well as to acetabular dysplasia at skeletal maturity is needed.

## Supplementary Information

**Additional file 1: Table 1.** a-d The statistical five-step hierarchic model examine if early life factors can predict four radiographic measurements (a-d) of acetabular dysplasia at 18-19 years of age. In total 2340 individuals were included for analysis. **Table 2.** Inverse probability weights (IPW) were applied in the final five-step model for each of the radiographic measurements, as a sensitivity analysis to account for the sampling from those from the original RCT who were invited to the maturity review (*n*=4469).

## Data Availability

The datasets used and/or analysed during the current study are available from the corresponding author upon reasonable request.

## Abbreviations

ADR: Acetabular depth-width ratio
AVN: Avascular necrosis
CE: Centre-edge angle
DDH: Developmental dysplasia of the hip
FHEI: Femoral head extrusion index
RCT: Randomized controlled trial
US: Ultrasound
