# Supplementary material for: Radiographic markers of hip dysplasia in young adults: predictive effect of factors in early life
Source: BMC Musculoskelet Disord. 2023 Feb 11;24:119. doi: 10.1186/s12891-023-06199-y (PMC9921356; doi:10.1186/s12891-023-06199-y)
Supplement: Supplementary file 1 — Additional file 1: Table 1. a-d The statistical five-step hierarchic model examine if early life factors can predict four radiographic measurements (a-d) of acetabular dysplasia at 18-19 years of age. In total 2340 individuals were included for analysis. Table 2. Inverse probability weights (IPW) were applied in the final five-step model for each of the radiographic measurements, as a sensitivity analysis to account for the sampling from those from the original RCT who were invited to the maturity review (n=4469). [file 12891_2023_6199_MOESM1_ESM.docx]

Table 1 a-d:

The statistical five-step hierarchic model examine if early life factors can predict four radiographic measurements (a-d) of acetabular dysplasia at 18-19 years of age. In total 2340 individuals were included for analysis.

Abbreviations: US ultrasound, Abd abduction; y years; AIC Akaike information criterion; ICC intra class correlations; CE center edge angle; FHEI femoral head extrusion index; ADR acetabular depth-width ratio

Table 1 a:

|  | Step 1 |  | Step 2 |  | Step 3 |  | Step 4 |  | Step 5 |  |
| --- | --- | --- | --- | --- | --- | --- | --- | --- | --- | --- |
| **Wiberg CE angle** | b (95% CI) | p | b (95% CI) | p | b (95% CI) | p | b (95% CI) | p | b (95% CI) | p |
| Male | 0 | ref | 0 | ref | 0 | ref | 0 | ref | 0 | ref |
| Female | **-1.25 (-1.71;-0.79)** | <0.001 | **-1.18 (-1.64;-0.71)** | <0.001 | **-1.16 (-1.63;-0.69)** | <0.001 | **-1.11 (-1.58;-0.65)** | <0.001 | **-1.11 (-1.57;-0.64)** | <0.001 |
| Birth length | 0.01 (-0.10;0.13) | 0.829 | 0.01 (-0.10;0.13) | 0.802 | 0.01 (-0.10;0.13) | 0.797 | 0.01 (-0.10;0.13) | 0.811 | 0.01 (-0.11;0.12) | 0.930 |
| Birth weight | 0.09 (-0.15;0.33) | 0.454 | 0.10 (-0.14;0.33) | 0.427 | 0.10 (-0.13;0.34) | 0.397 | 0.10 (-0.13;0.34) | 0.392 | 0.08 (-0.16;0.32) | 0.515 |
| Family history | -0.18 (-1.06;0.70) | 0.692 | -0.13 (-1.01;0.74) | 0.767 | -0.09 (-0.98;0.79) | 0.836 | 0.01 (-0.87;0.89) | 0.986 | -0.01 (-0.89;0.87) | 0.985 |
| No Fam.history | 0 | Ref | 0 | ref | 0 | ref | 0 | ref | 0 | Ref |
| Normal cephalic birth | 0 | Ref | 0 | ref | 0 | ref | 0 | ref | 0 | ref |
| Breech | **-1.41 (-2.42;-0.40)** | 0.006 | **-1.38 (-2.39;-0.37)** | 0.008 | **-1.34 (-2.36;-0.32)** | 0.010 | **-1.33 (-2.35;-0.31)** | 0.010 | **-1.32 (-2.34;-0.31)** | 0.011 |
| Uncommon cephalic | -0.51 (-1.65;0.62) | 0.373 | -0.49 (-1.62;0.64) | 0.396 | -0.49 (-1.62;0.64) | 0.395 | -0.48 (-1.61;0.64) | 0.401 | -0.46 (-1.59;0.66) | 0.421 |
| Right side | 0 | ref | 0 | ref | 0 | Ref | 0 | ref | 0 | ref |
| Left side | **0.62 (0.43;0.81)** | <0.001 | **0.65 (0.45;0.84)** | <0.001 | **0.64 (0.45;0.83)** | <0.001 | **0.64 (0.45;0.83)** | <0.001 | **0.64 (0.45;0.83)** | <0.001 |
| Clinically stable hips | 0 | ref | 0 | ref | 0 | ref | 0 | ref | 0 | Ref |
| Clinical hip instability | -0.62 (-1.41;0.16) | 0.117 | -0.55 (-1.35;0.26) | 0.182 | -0.51 (-1.32;0.30) | 0.218 | -0.52 (-1.32;0.29) | 0.209 | -0.52 (-1.33;0.28) | 0.204 |
| Alpha angle US |  |  | **0.06 (0.03;0.09)** | <0.001 | **0.06 (0.02;0.09)** | 0.001 | **0.06 (0.02;0.09)** | 0.001 | **0.06 (0.02;0.09)** | 0.001 |
| Hip instab. US |  |  | 0.34 (-0.02;0.70) | 0.060 | **0.39 (0.01;0.77)** | 0.042 | **0.39 (0.01;0.76)** | 0.043 | **0.38 (0.01;0.76)** | 0.045 |
| No Abd Treatment |  |  |  |  | 0 | ref | 0 | ref | 0 | ref |
| Abd Treatment |  |  |  |  | -0.41 (-1.41;0.58) | 0.416 | -0.46 (-1.46;0.53) | 0.359 | -0.46 (-1.46;0.53) | 0.361 |
| No Late Abd Treatm |  |  |  |  |  |  | 0 | ref | 0 | ref |
| Late Abd Treatment |  |  |  |  |  |  | **-3.84 (-6.37;-1.30)** | 0.003 | **-3.83 (-6.37;-1.30)** | 0.003 |
| Slope height 0-12 y |  |  |  |  |  |  |  |  | -0.08 (-0.17;0.01) | 0.075 |
| Slope weight 0-12 y |  |  |  |  |  |  |  |  | 0.38 (-0.01;0.78) | 0.056 |
| Slope BMI 0-12 y |  |  |  |  |  |  |  |  | -0.05 (-0.38;0.28) | 0.771 |
| AIC | 28488.7 |  | 28480.2 |  | 28481.6 |  | 28474.8 |  | 28475.4 |  |

Crude AIC=28567.5

Crude ICC=0.69 (95% CI: 0.67;0.71)

Table 1 b:

|  | Step 1 |  | Step 2 |  | Step 3 |  | Step 4 |  | Step 5 |  |
| --- | --- | --- | --- | --- | --- | --- | --- | --- | --- | --- |
| **FHEI** | b (95% CI) | P | b (95% CI) | p | b (95% CI) | p | b (95% CI) | p | b (95% CI) | P |
| Male | 0 | ref | 0 | ref | 0 | ref | 0 | ref | 0 | ref |
| Female | -0.26 (-0.75;0.23) | 0.302 | -0.20 (-0.69;0.30) | 0.433 | -0.15 (-0.64;0.35) | 0.559 | -0.08 (-0.58;0.41) | 0.736 | -0.09 (-0.59;0.40) | 0.709 |
| Birth length | 0.05 (-0.07;0.17) | 0.418 | 0.05 (-0.07;0.17) | 0.406 | 0.05 (-0.07;0.17) | 0.398 | 0.05 (-0.07;0.17) | 0.410 | 0.04 (-0.08;0.16) | 0.538 |
| Birth weight | 0.06 (-0.19;0.31) | 0.659 | 0.06 (-0.19;0.31) | 0.632 | 0.08 (-0.17;0.33) | 0.532 | 0.08 (-0.17;0.33) | 0.525 | 0.04 (-0.21;0.30) | 0.754 |
| Family history | -0.35 (-1.29;0.58) | 0.459 | -0.32 (-1.25;0.62) | 0.505 | -0.21 (-1.14;0.73) | 0.666 | -0.07 (-1.01;0.87) | 0.883 | -0.1 (-1.04;0.83) | 0.828 |
| No Fam.history | 0 | ref | 0 | ref | 0 | ref | 0 | ref | 0 | ref |
| Normal cephalic birth | 0 | ref | 0 | ref | 0 | ref | 0 | ref | 0 | Ref |
| Breech | -1.04 (-2.12;0.04) | 0.059 | -1.03 (-2.11;0.05) | 0.061 | -0.92 (-2.00;0.16) | 0.096 | -0.91 (-1.99;0.17) | 0.099 | -0.96 (-2.04;0.12) | 0.081 |
| Uncommon cephalic | -0.61 (-1.81;0.59) | 0.321 | -0.59 (-1.80;0.61) | 0.332 | -0.60 (-1.80;0.60) | 0.329 | -0.59 (-1.78;0.61) | 0.336 | -0.56 (-1.75;0.63) | 0.357 |
| Right side | 0 | ref | 0 | ref | 0 | ref | 0 | ref | 0 | Ref |
| Left side | **1.17 (0.96;1.39)** | <0.001 | **1.20 (0.97;1.42)** | <0.001 | **1.18 (0.95;1.40)** | <0.001 | **1.18 (0.95;1.40)** | <0.001 | **1.18 (0.95;1.40)** | <0.001 |
| Clinically stable hips | 0 | ref | 0 | ref | 0 | ref | 0 | ref | 0 | ref |
| Clinical hip instability | **-1.14 (-2.01;-0.27)** | 0.010 | **-1.11 (-2.01;-0.21)** | 0.015 | **-0.99 (-1.89;-0.09)** | 0.032 | **-1.00 (-1.91;-0.10)** | 0.029 | **-1.01 (-1.91;-0.11)** | 0.028 |
| Alpha angle US |  |  | **0.06 (0.02;0.09)** | 0.001 | **0.05 (0.02;0.09)** | 0.004 | **0.05 (0.02;0.09)** | 0.004 | **0.05 (0.02;0.09)** | 0.004 |
| Hip instab. US |  |  | **0.43 (0.03;0.83)** | 0.033 | **0.58 (0.16;1.00)** | 0.007 | **0.57 (0.16;0.99)** | 0.007 | **0.58 (0.16;1.00)** | 0.007 |
| No Abd Treatment |  |  |  |  | 0 | ref | 0 | ref | 0 | ref |
| Abd Treatment |  |  |  |  | **-1.18 (-2.25;-0.11)** | 0.030 | **-1.25 (-2.32;-0.18)** | 0.021 | **-1.25 (-2.31;-0.18)** | 0.022 |
| No Late Abd Treatm |  |  |  |  |  |  | 0 | ref | 0 | ref |
| Late Abd Treatment |  |  |  |  |  |  | **-5.15 (-7.84;-2.46)** | <0.001 | **-5.1 (-7.78;-2.41)** | <0.001 |
| Slope height 0-12 y |  |  |  |  |  |  |  |  | -0.08 (-0.17;0.02) | 0.100 |
| Slope weight 0-12 y |  |  |  |  |  |  |  |  | **0.53 (0.11;0.94)** | 0.013 |
| Slope BMI 0-12 y |  |  |  |  |  |  |  |  | -0.01 (-0.36;0.34) | 0.958 |
| AIC | 29412.4 |  | 29406.0 |  | 29403.3 |  | 29391.2 |  | 29381.5 |  |

Crude AIC=29529.3

Crude ICC=0.63 (95% CI:0.61;0.66)

Table 1 c:

|  | Step 1 |  | Step 2 |  | Step 3 |  | Step 4 |  | Step 5 |  |
| --- | --- | --- | --- | --- | --- | --- | --- | --- | --- | --- |
| **ADR** | b (95% CI) | p | b (95% CI) | p | b (95% CI) | p | b (95% CI) | p | b (95% CI) | p |
| Male | 0 | ref | 0 | ref | 0 | Ref | 0 | ref | 0 | ref |
| Female | **2.93 (0.22;5.65)** | 0.034 | **3.08 (0.35;5.82)** | 0.027 | **3.21 (0.46;5.95)** | 0.022 | **3.43 (0.68;6.18)** | 0.015 | **3.51 (0.76;6.27)** | 0.012 |
| Birth length | -0.24 (-0.91;0.43) | 0.481 | -0.24 (-0.90;0.43) | 0.482 | -0.24 (-0.90;0.43) | 0.487 | -0.24 (-0.91;0.42) | 0.477 | -0.24 (-0.91;0.42) | 0.472 |
| Birth weight | 0.07 (-1.32;1.46) | 0.919 | 0.08 (-1.31;1.47) | 0.906 | 0.13 (-1.26;1.52) | 0.856 | 0.13 (-1.26;1.52) | 0.851 | 0.1 (-1.32;1.53) | 0.887 |
| Family history | -3.39 (-8.56;1.78) | 0.198 | -3.31 (-8.48;1.86) | 0.209 | -3.04 (-8.24;2.16) | 0.252 | -2.57 (-7.78;2.64) | 0.334 | -2.52 (-7.73;2.69) | 0.343 |
| No Fam.history | 0 | Ref | 0 | ref | 0 | ref | 0 | ref | 0 | ref |
| Normal cephalic birth | 0 | Ref | 0 | ref | 0 | ref | 0 | ref | 0 | ref |
| Breech | **-10.64 (-16.60;-4.68)** | <0.001 | **-10.68 (-16.65;-4.71)** | <0.001 | **-10.39 (-16.39;-4.39)** | 0.001 | **-10.35 (-16.34;-4.36)** | 0.001 | **-10.13 (-16.13;-4.12)** | 0.001 |
| Uncommon cephalic | -3.41 (-10.07;3.25) | 0.316 | -3.40 (-10.06;3.26) | 0.317 | -3.41 (-10.06;3.25) | 0.316 | -3.37 (-10.02;3.28) | 0.320 | -3.39 (-10.04;3.26) | 0.318 |
| Right side | 0 | Ref | 0 | Ref | 0 | ref | 0 | Ref | 0 | ref |
| Left side | **3.17 (2.09;4.25)** | <0.001 | **3.21 (2.12;4.30)** | <0.001 | **3.17 (2.07;4.26)** | <0.001 | **3.17 (2.07;4.26)** | <0.001 | **3.17 (2.07;4.27)** | <0.001 |
| Clinically stable hips | 0 | ref | 0 | Ref | 0 | Ref | 0 | ref | 0 | Ref |
| Clinical hip instability | -3.22 (-7.70;1.26) | 0.159 | -3.36 (-7.96;1.25) | 0.153 | -3.10 (-7.73;1.53) | 0.190 | -3.15 (-7.78;1.48) | 0.182 | -3.17 (-7.80;1.47) | 0.180 |
| Alpha angle US |  |  | 0.18 (-0.01;0.37) | 0.057 | 0.17 (-0.02;0.36) | 0.078 | 0.17 (-0.02;0.36) | 0.080 | 0.17 (-0.02;0.36) | 0.079 |
| Hip instab. US |  |  | 1.67 (-0.39;3.72) | 0.112 | 1.98 (-0.18;4.14) | 0.072 | 1.97 (-0.19;4.12) | 0.074 | 1.94 (-0.22;4.10) | 0.078 |
| No Abd Treatment |  |  |  |  | 0 | ref | 0 | Ref | 0 | Ref |
| Abd Treatment |  |  |  |  | -2.84 (-8.67;2.98) | 0.339 | -3.09 (-8.91;2.74) | 0.299 | -3.05 (-8.88;2.78) | 0.305 |
| No Late Abd Treatm |  |  |  |  |  |  | 0 | Ref | 0 | Ref |
| Late Abd Treatment |  |  |  |  |  |  | **-17.97 (-32.92;-3.03)** | 0.018 | **-18.13 (-33.08;-3.19)** | 0.017 |
| Slope height 0-12 y |  |  |  |  |  |  |  |  | -0.07 (-0.60;0.45) | 0.788 |
| Slope weight 0-12 y |  |  |  |  |  |  |  |  | 0.25 (-2.07;2.57) | 0.834 |
| Slope BMI 0-12 y |  |  |  |  |  |  |  |  | -0.55 (-2.50;1.40) | 0.581 |
| AIC | 44898.9 |  | 44898.86 |  | 44900.0 |  | 44896.4 |  | 44901.2 |  |

Crude AIC=44960.8

Crude ICC=0.71 (95% CI:0.69;0.73)

Table 1 d:

|  | Step 1 |  | Step 2 |  | Step 3 |  | Step 4 |  | Step 5 |  |
| --- | --- | --- | --- | --- | --- | --- | --- | --- | --- | --- |
| **Sharp** | b (95% CI) | p | b (95% CI) | p | b (95% CI) | p | b (95% CI) | p | b (95% CI) | p |
| Male | 0 | ref | 0 | ref | 0 | ref | 0 | ref | 0 | ref |
| Female | **2.05 (1.81;2.29)** | <0.001 | **1.99 (1.74;2.23)** | <0.001 | **1.97 (1.73;2.22)** | <0.001 | **1.96 (1.72;2.20)** | <0.001 | **1.95 (1.71;2.20)** | <0.001 |
| Birth length | -0.04 (-0.10;0.02) | 0.192 | -0.04 (-0.10;0.02) | 0.162 | -0.04 (-0.10;0.02) | 0.161 | -0.04 (-0.10;0.02) | 0.164 | -0.04 (-0.10;0.02) | 0.229 |
| Birth weight | -0.06 (-0.19;0.06) | 0.305 | -0.07 (-0.19;0.05) | 0.273 | -0.08 (-0.20;0.05) | 0.230 | -0.08 (-0.20;0.05) | 0.228 | -0.06 (-0.19;0.06) | 0.329 |
| Family history | -0.11 (-0.57;0.35) | 0.637 | -0.15 (-0.61;0.31) | 0.525 | -0.19 (-0.65;0.28) | 0.430 | -0.22 (-0.68;0.24) | 0.356 | -0.21 (-0.67;0.25) | 0.380 |
| No Fam.history | 0 | ref | 0 | ref | 0 | ref | 0 | ref | 0 | ref |
| Normal cephalic birth | 0 | ref | 0 | ref | 0 | ref | 0 | ref | 0 | ref |
| Breech | 0.53 (-0.01;1.06) | 0.053 | 0.48 (-0.05;1.02) | 0.076 | 0.45 (-0.08;0.99) | 0.096 | 0.45 (-0.08;0.99) | 0.098 | 0.44 (-0.09;0.98) | 0.106 |
| Uncommon cephalic | 0.23 (-0.36;0.82) | 0.441 | 0.20 (-0.39;0.79) | 0.515 | 0.20 (-0.39;0.79) | 0.507 | 0.20 (-0.39;0.79) | 0.512 | 0.18 (-0.41;0.77) | 0.543 |
| Right side | 0 | ref | 0 | ref | 0 | ref | 0 | ref | 0 | ref |
| Left side | -0.03 (-0.20;0.13) | 0.693 | -0.07 (-0.23;0.10) | 0.430 | -0.06 (-0.22;0.11) | 0.499 | -0.06 (-0.22;0.11) | 0.499 | -0.06 (-0.22;0.11) | 0.499 |
| Clinically stable hips | 0 | ref | 0 | Ref | 0 | ref | 0 | ref | 0 | ref |
| Clinical hip instability | 0.15 (-0.38;0.68) | 0.583 | -0.08 (-0.64;0.47) | 0.767 | -0.14 (-0.71;0.42) | 0.617 | -0.14 (-0.70;0.42) | 0.630 | -0.13 (-0.69;0.43) | 0.646 |
| Alpha angle US |  |  | **-0.03 (-0.05;-0.01)** | 0.004 | **-0.03 (-0.05;-0.01)** | 0.008 | **-0.03 (-0.05;-0.01)** | 0.008 | **-0.03 (-0.05;-0.01)** | 0.009 |
| Hip instab. US |  |  | 0.02 (-0.22;0.25) | 0.896 | -0.06 (-0.31;0.20) | 0.653 | -0.06 (-0.31;0.20) | 0.663 | -0.05 (-0.31;0.20) | 0.690 |
| No Abd Treatment |  |  |  |  | 0 | ref | 0 | ref | 0 | Ref |
| Abd Treatment |  |  |  |  | 0.43 (-0.13;0.99) | 0.133 | 0.44 (-0.11;1.00) | 0.119 | 0.44 (-0.11;1.00) | 0.119 |
| No Late Abd Treatm |  |  |  |  |  |  | 0 | ref | 0 | Ref |
| Late Abd Treatment |  |  |  |  |  |  | 1.20 (-0.12;2.53) | 0.075 | 1.21 (-0.12;2.53) | 0.074 |
| Slope height 0-12 y |  |  |  |  |  |  |  |  | **0.06 (0.01;0.11)** | 0.012 |
| Slope weight 0-12 y |  |  |  |  |  |  |  |  | **-0.26 (-0.46;-0.05)** | 0.015 |
| Slope BMI 0-12 y |  |  |  |  |  |  |  |  | 0.03 (-0.15;0.20) | 0.764 |
| AIC | 24767.4 |  | 24758.4 |  | 24758.2 |  | 24757.0 |  | 24756.8 |  |

Crude AIC=25055.7

Crude ICC=0.39 (95% CI: 0.35;0.42)

Table 2:

Inverse probability weights (IPW) were applied in the final five-step model for each of the radiographic measurements, as a sensitivity analysis to account for the sampling from those from the original RCT who were invited to the maturity review (n=4469).

|  |  | **Wiberg** |  |  | **FHEI** |  | **ADR** |  | **Sharp** |  |
| --- | --- | --- | --- | --- | --- | --- | --- | --- | --- | --- |
|  |  | b (95% CI) | p |  | b (95% CI) | p | b (95% CI) | p | b (95% CI) | p |
| Male |  | 0 | . |  | 0 | . | 0 | . | 0 | . |
| Female |  | **-1.12 (-1.59;-0.65)** | <0.001 |  | -0.12 (-0.61;0.37) | 0.642 | **3.46 (0.74;6.18)** | 0.013 | **1.95 (1.71;2.20)** | <0.001 |
| Birth length |  | 0.01 (-0.08;0.11) | 0.806 |  | 0.03 (-0.06;0.12) | 0.503 | -0.16 (-0.82;0.50) | 0.640 | -0.04 (-0.09;0.02) | 0.170 |
| Birth weight |  | 0.08 (-0.08;0.25) | 0.316 |  | 0.05 (-0.11;0.22) | 0.533 | -0.02 (-1.59;1.54) | 0.975 | -0.06 (-0.21;0.08) | 0.391 |
| Family history |  | 0.12 (-0.72;0.96) | 0.779 |  | -0.03 (-0.95;0.89) | 0.946 | -1.45 (-6.43;3.52) | 0.567 | -0.18 (-0.64;0.27) | 0.429 |
| No Fam.history |  | 0 | . |  | 0 | . | 0 | . | 0 | . |
| Normal cephalic birth |  | 0 | . |  | 0 | . | 0 | . | 0 | . |
| Breech |  | **-1.28 (-2.29;-0.28)** | 0.012 |  | -0.86 (-1.91;0.20) | 0.113 | **-9.9 (-15.82;-3.98)** | 0.001 | 0.37 (-0.16;0.90) | 0.174 |
| Uncommon cephalic |  | -0.52 (-1.64;0.59) | 0.358 |  | -0.61 (-1.73;0.50) | 0.282 | -3.32 (-8.85;2.21) | 0.239 | 0.22 (-0.39;0.83) | 0.486 |
| Right side |  | 0 | . |  | 0 | . | 0 | . | 0 | . |
| Left side |  | **0.66 (0.47;0.86)** | <0.001 |  | **1.13 (0.91;1.36)** | <0.001 | **3.04 (1.93;4.16)** | <0.001 | -0.06 (-0.23;0.11) | 0.462 |
| Clinically stable hips |  | 0 | . |  | 0 | . | 0 | . | 0 | . |
| Clinical hip instability |  | -0.63 (-1.42;0.16) | 0.117 |  | **-1.11 (-1.98;-0.25)** | 0.012 | -3.02 (-7.50;1.45) | 0.186 | -0.06 (-0.60;0.48) | 0.831 |
| Alpha angle US |  | **0.05 (0.02;0.09)** | 0.005 |  | **0.05 (0.01;0.08)** | 0.016 | 0.16 (-0.02;0.33) | 0.078 | -0.02 (-0.05;0.00) | 0.072 |
| Hip instab. US |  | 0.35 (-0.04;0.74) | 0.075 |  | **0.53 (0.12;0.94)** | 0.011 | 1.62 (-0.47;3.71) | 0.130 | -0.02 (-0.31;0.27) | 0.876 |
| No Abd Treatment |  | 0 | . |  | 0 | . | 0 | . | 0 | . |
| Abd Treatment |  | -0.47 (-1.57;0.63) | 0.400 |  | **-1.22 (-2.36;-0.09)** | 0.035 | -3.21 (-9.31;2.89) | 0.303 | 0.42 (-0.15;0.99) | 0.153 |
| No Late Abd Treatm |  | 0 | . |  | 0 | . | 0 | . | 0 | . |
| Late Abd Treatment |  | **-3.86 (-6.09;-1.63)** | 0.001 |  | **-5.08 (-7.52;-2.64)** | <0.001 | **-18.43 (-36.55;-0.31)** | 0.046 | **1.20 (0.14;2.25)** | 0.026 |
| Slope height 0-12 y |  | -0.08 (-0.17;0.01) | 0.067 |  | -0.07 (-0.17;0.02) | 0.120 | -0.12 (-0.67;0.42) | 0.663 | **0.06 (0.01;0.11)** | 0.012 |
| Slope weight 0-12 y |  | 0.33 (-0.12;0.77) | 0.149 |  | 0.45 (-0.03;0.93) | 0.064 | 0.13 (-2.64;2.90) | 0.926 | **-0.28 (-0.49;-0.07)** | 0.009 |
| Slope BMI 0-12 y |  | -0.01 (-0.37;0.34) | 0.936 |  | 0.03 (-0.36;0.42) | 0.881 | -0.33 (-2.61;1.95) | 0.774 | 0.05 (-0.11;0.22) | 0.548 |
